# Pharmacognostic Study on *Elsholtzia ciliata* (Thumb.) Hyl: Anatomy, Phytochemistry and Pharmacological Activities

**DOI:** 10.3390/ph14111152

**Published:** 2021-11-12

**Authors:** Qian Zhang, Niara Moura Porto, Carolina Carvalho Guilhon, Thais Biondino Sardella Giorno, Daniela Sales Alviano, Maria de Fatima Agra, Patricia Dias Fernandes, Fabio Boylan

**Affiliations:** 1 School of Pharmacy and Pharmaceutical Sciences, Trinity Biomedical Sciences Institute, Trinity College Dublin, D02 PN40 Dublin, Ireland; 2 Intelligent Sensing and AI Center, National University of Singapore (Chongqing) Research Institute, Chongqing 401123, China; 3 Laboratory of Taxonomy and Pharmacobotany, Federal University of Paraíba, João Pessoa 58051-970, Brazil; niaraporto@gmail.com (N.M.P.); agramf@ltf.ufpb.br (M.d.F.A.); 4 Laboratory of Pharmacology of Pain and Inflammation, Institute of Biomedical Sciences, Federal University of Rio de Janeiro, Av. Carlos Chagas Filho 373, bloco F. Prédio novo ICB, Rio de Janeiro 21941-901, Brazil; carolguilhon@gmail.com (C.C.G.); thais.sardella.farma@hotmail.com (T.B.S.G.); patricia.dias@icb.ufrj.br (P.D.F.); 5 Department of Microbiology, Federal University of Rio de Janeiro, Rio de Janeiro 21941-901, Brazil; danialviano@gmail.com

**Keywords:** *Elsholtzia ciliata*, botanical identification, chemical analysis, anti-inflammatory activity, quality control, Traditional Chinese Medicine

## Abstract

*Elsholtzia ciliata* (Thunb.) Hyl, family Lamiaceae, is an important and popular anti-bacterial and anti-inflammatory Traditional Chinese Medicine (TCM). However, there are limited scientific studies on its anatomy and pharmacological activities. Moreover, the information of chemical constituents in relation to its non-volatile constituents are still missing. The current study aimed to evaluate the anatomic, pharmacological and phytochemical profile of *Elsholtzia ciliata*, providing means for the quality control of this herbal drug. The methodology designed for this study included the preparation of anatomic sections and their description, extraction, chromatography, structural elucidation of isolated compounds by NMR techniques and their quantification by HPLC using pharmacological assays (Formalin, hot plate, DPPH, antimicrobial-Gram positive, Gram Negative and fungus, and MTT assays) to confirm the activities described for this species. Results of the anatomic study are aligned with the pattern expected for plants belonging to the Lamiaceae family; Ursolic acid and Oroxylin were isolated from this plant species. The findings observed in this study indicate that *Elsholtzia ciliata* possess anti-inflammatory, antinociceptive, antioxidant, antimicrobial and anticancer activities. The chemical compounds isolated from its leaves and the anatomy profile of its parts provide the basis for further quality control for this plant.

## 1. Introduction

For thousands of years, plants have been a source of medicine; people relied on nature to supply their basic needs, including their medicines. Several traditional medicinal systems such as Traditional Chinese Medicine, Indian Ayurvedic and the ancient Greek and Romans systems [1] have formed and made a substantial contribution to the development of natural medicine [2]. As a typical ethnomedicinal approach, Traditional Chinese Medicine (TCM) is one of a few ancient medicinal systems still preserved to date. It has gained more acceptance and recognition by other countries due to its affordability, reliable efficacy, local availability and fewer described side effects [3]. However, the indistinct effects, poor quality control, and the differences concerning the modern medical system restrict its global use.

*Elsholtzia ciliata* (Thunb.) Hyl. (EC), family Lamiaceae is a plant widely used in TCM as an anti-bacterial and anti-inflammatory medicine [4]. EC occurs widely in Jiangxi, Yunnan Province of China and throughout India, Korea and Europe. EC is an herbaceous plant measuring 30–50 cm in height with thick leaves, an oval leaflet, a growing villus and brownish oblong berry-shaped seeds. The flower is pink and usually contains 3–4 pistils in which the lower lobe is lower than the upper lobe [5]. EC has been used to treat a range of diseases, such as colds, fever, diarrhea, dysentery, digestion disorders, vomiting, strokes and oedema for thousands of years in folk medicine in China [4]. There are reports of EC as playing a role in antimicrobial, antioxidant, antinociceptive, anti-inflammatory, febrifuge, sedative, anticancer and antitumor activities [6,7]. However, thus far, there has been no testing for these activities but the essential oil of this plant has had its antimicrobial activity evaluated a few times [5]. A few studies have reported the chemistry of EC, most of them about the chemical composition of the essential oil, and hence, more studies are required to evaluate the non-volatile chemical composition. The main chemical compounds for EC are volatile oil constituents, flavonoids, steroids and triterpenes [8].

This study dealt with EC’s anatomy, non-volatile composition and pharmacological properties. The study aimed at providing information for the quality control (Botany and Chemistry) of this herbal drug, traditionally used in China for many centuries.

## 2. Results

### 2.1. Anatomic Study

**Figure 1:**
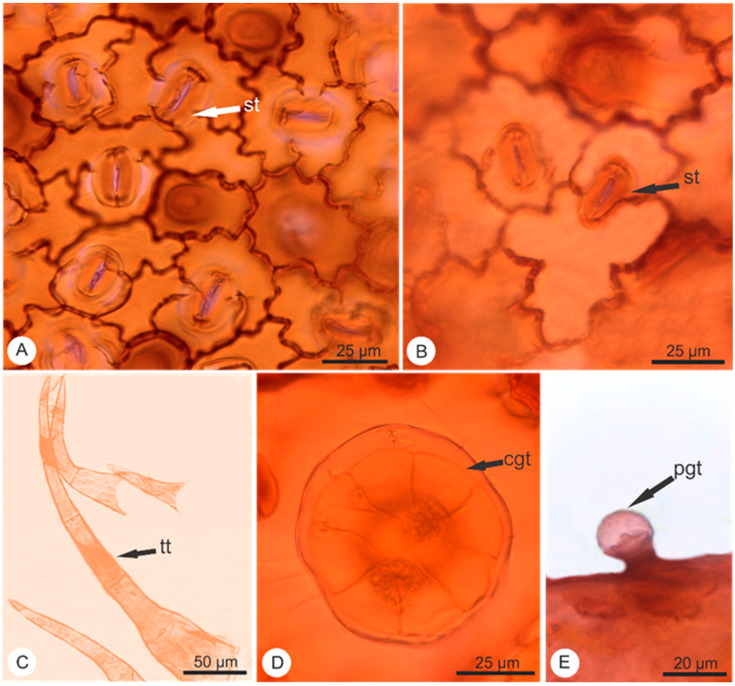
Light micrographs of epidermis of *Elsholtzia ciliata* (Thunb.) Hyl. (**A**) Adaxial surface with stomata anisocytic and diacytic; (**B**) Abaxial surface with detail of diacytic stomata. (**C**) Detail of multicellular tector trichome; (**D**) Detail of capitate glandular trichome; (**E**) Detail of peltate glandular trichome. Legend: capitate glandular trichome (cgt); peltate glandular trichome (pgt); stomata (st); multicellular tector trichome (tt).

**Figure 2:**
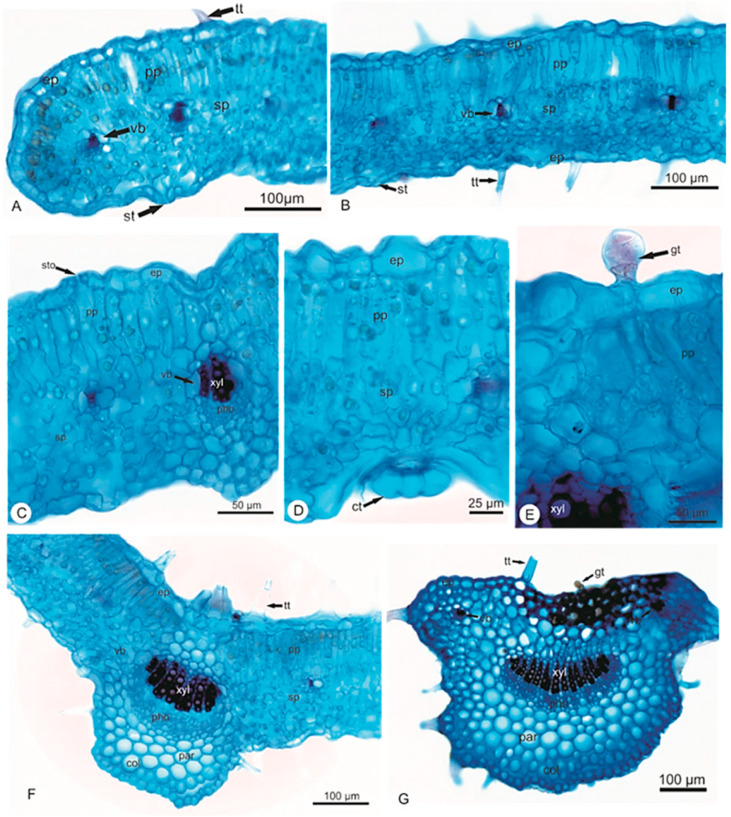
Cross sections of leaf of *Elsholtzia ciliata* (Thunb.) Hyl. (**A**) Margin with circular contour, presence of multicellular tector trichome; (**B**) Mesophyll dorsiventral with small collateral vascular bundles; (**C**) Detail of mesophyll with uniseriate epidermis, uniseriate palisade parenchyma and several layers of spongy parenchyma; (**D**) Detail of mesophyll with capitate glandular trichome present in the abaxial surface; (**E**) Detail of glandular trichome present in the adaxial surface; (**F**) Midrib has concave–convex contour and one collateral vascular bundle; (**G**) Petiole with plan-convex contour, with one larger bundle in the central portion and two smaller bundles. Legend: collenchyma (col); capitate glandular trichome (ct); epidermis (ep); peltate glandular trichome (gt); parenchyma (par); palisade parenchyma (pp); phloem (pho); spongy parenchyma (sp); stomata (st); tector trichome (tt); vascular bundle (vb); xylem (xyl); Stomata (sto).

**Figure 3:**
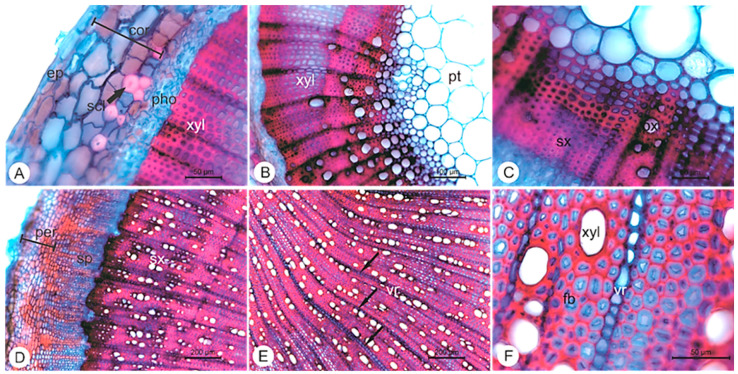
Cross sections of stem of *Elsholtzia ciliata* (Thunb.) Hyl. (**A**) Stem primary growth, with uniseriate epidermis; (**B**) Detail of vascular tissue and a parenchyma pith; (**C**) Detail of xylem; (**D**) Stem secondary growth, with secondary vascular tissue and several layers of periderm; (**E**) Detail of secondary vascular tissue divided by parenchyma rays; (**F**) Detail of xylem tissue with fibers. Legend: cortex (cor); epidermis (ep); fibers (fb); periderm (per); phloem (pho); pith (pt); primary xylem (px); sclereids (scl); secondary phloem (sp); secondary xylem (sx); vascular ray (vr); xylem (xyl).

Transverse sections (TS) were prepared using the leaves and stems of *Elsholtzia ciliata*. Slides with the TS were then observed under the microscope resulting in the following findings (Figure 1, Figure 2 and Figure 3):

The leaf blade of *Elsholtzia ciliata* proved to be amphistomatic with diacytic and anisocytic stomata. The diacytic type predominated the front view. The epidermis showed sinuous anticlinal walls in both adaxial and abaxial surfaces (Figure 1A,B). The analysis of the epidermis by light microscopy (LM) revealed a smooth cuticle surface with three types of trichomes: simple multicellular trichome (Figure 1C), capitate glandular trichome (Figure 1D), and peltate glandular trichome (Figure 1E), being observed on both surfaces. The simple multicellular trichomes are non-glandular and consist of 1–6 cells with an aduncous contour at the end, the capitate glandular has eight cells in the round multicellular secretory head and a monocellular stalk. The peltate glandular type contains a basal epidermal cell with a round multicellular secretory head and a monocellular stalk.

In the cross-section, the leaf margin is curved towards the abaxial surface with a uniseriate epidermis. Palisade parenchyma follows with small collateral vascular bundles surrounded by 2–4 layers of spongy parenchyma cells (Figure 2A). The mesophyll is dorsiventral with 1-stratified palisade parenchyma (Figure 2B). Stomata showed the same level of epidermal cells according to its arrangement (Figure 2C). Several small collateral vascular bundles are distributed throughout the mesophyll, surrounded by a parenchymatic sheath. Three types of trichomes are present: simple multicellular, capitate glandular (Figure 2D) and peltate glandular (Figure 2E).

The midrib is of a concave–convex shape, with epidermal cells of smaller sizes in the region of the leaf blade. Subjacent to the epidermis (on the adaxial side) one to two layers of lacunar collenchyma are present. Fundamental parenchyma follows. The vascular tissue is collateral and arranged in a single bundle (Figure 2F).

In the cross-section, the petiole shows a concave–convex contour, forming two ribs (Figure 2G). The epidermis is uniseriate (with covering and glandular trichomes—similar to those described for the leaf blade) on the epidermal surface. The cortex is composed of discontinuous lacunar collenchyma. Below the collenchyma, there are about three to four layers of homogeneous chlorenchyma. The vascular system is collateral, arranged in three vascular bundles, having a central, larger bundle with an open arch and two other auxiliary, smaller and cylindrical bundles.

The stem is four prismatic in shape. In primary structure, the stem shows a uniseriate epidermis with tabular cells (Figure 3A). The cortex has 2–3 layers of parenchyma cells and discontinuous layers of lacunar collenchyma. In collenchyma of more internal cells, collenchyma undergo sclerification differing in fiber. The vascular tissue is arranged in single cylinder collateral surrounding the parenchyma pith (Figure 3B,C).

The secondary growth is the development of secondary vascular tissue and periderm. The cross-sections showed a 4–8 layered periderm, with rectangular cells, with compact phellem formed by uniform, thickened cells and phelloderm containing about 1–3 layers of parenchyma cells (Figure 3D). The collateral vascular tissue has diffused and poriferous vessel elements with circle elliptical contour, having two different diameters—wide and narrow (Figure 3E). The rays are generally composed of uniseriate parenchyma (with some homogeneous biseriate rays) consisting of square cells (Figure 3F). The pith is formed by parenchyma cells in the central portion.

This study provides important anatomy and morphology information of *Elsholtzia ciliata* (Thunb.) Hyl. Although some basic descriptions in Chinese Pharmacopeia [9], as well as a similar anatomic study of Korean species were previously performed [10,11], the current work provides more specific and systematic anatomic characteristics for this species.

The current anatomic study shows that the stomata simultaneously possess amphistomatic leaves with diacytic and anisocytic types (and a predominance for the diacytic type); the difference when compared to the Chinese Pharmacopoeia is that a few anisocytic stomata were also observed. The current study also showed sinuous anticlinal walls, which is in accordance with the Chinese Pharmacopoeia. The trichomes revealed similar features, the non-glandular trichome contains 1–6 cells while glandular trichome includes capitate and peltate. The mature capitate glandular trichome consisted of 8 cells in the head and a monocellular stalk. The peltate glandular trichome is not mentioned in the Chinese Pharmacopoeia but is a common characteristic found for this species, and also common in another species—*Elsholtzia stauntoni* Benth [12].

We also performed the leaf and stem cross-section comparisons in the current study and the other four studied species of the Elsholtzia genus: *Elsholtzia pilosa* (Benth.) Benth. *Elsholtzia penduliflora* W.W. Sm, *Elsholtzia heterophylla* Diels and *Elsholtzia kachinensis* Prain.

The comparison of the leaf cross-section between five species showed that all of them possessed the same characteristics with uniseriate epidermal cells on the upper and lower surfaces, the same collateral vascular bundles, 2–4 layer spongy parenchyma and presence of collenchyma on both surfaces. Meanwhile, trichomes and stomata differ among the five species. All of them contain glandular and non-glandular trichomes. Capitate and Peltate glandular trichomes were observed in *Elsholtzia ciliata* cross-sections. For *Elsholtzia ciliata* and *Elsholtzia heterophylla* Diels, they both have monocellular and multicellular trichomes, while *Elsholtzia penduliflora* W.W. Sm and *Elsholtzia kachinensis* Prain only contain multicellular trichomes. In addition, *Elsholtzia pilosa* (Benth.) Benth contains unique ramose non-glandular trichome, which could be used for its identification. The stomata type is normally one of diacytic and anisocytic and sometimes both, while some other species in this genus present anomocytic stomata [13].

The stem cross-section was also compared for these five species. The epidermis and collenchyma possessed the same characteristic. *Elsholtzia kachinensis* Prain is a different species with a circular stem cross-section contour and the absence of secondary growth structures. The main reason for this characteristic is that it is some sort of helophyte, without developed collenchyma and secondary growth structure [10].

The characteristics of the contour and vascular bundles are valuable for identification in the stem cross-section of different species.

### 2.2. Structure Elucidation of Isolated Compounds

#### 2.2.1. Unknown Precipitate Analysis

At the extraction procedure, a large quantity of a light green solid precipitated during the preparation of the hexane and dichloromethane fractions. Such large quantity of solid precipitate is necessary and worth considering for unknown precipitate analysis. This solid material (compound **1**) was then washed with hexane and dichloromethane to remove extract impurities and sent for NMR analysis.

**Figure 4:**
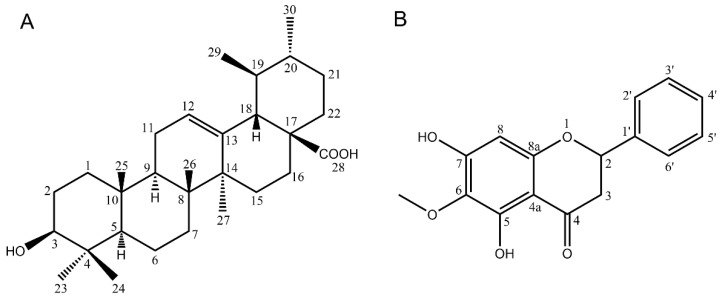
Proposed chemical structure of compound **1** (**A**) Ursolic acid and compound **2** (**B**) Oroxylin A.

The white precipitate had a melting point of 283–285 °C; this data taken together with NMR results suggested it to be ursolic acid (Figure 4A). The chemical formula of ursolic acid (UA) is C_30_H_48_O_3_ (molecular weight = 456). The 1H-NMR spectrum of UA showed the characteristic signals to be the pentacyclic triterpene ursolic acid [14]. This structure elucidation is also in agreement with the literature data described by Seebacher et al. [15].

Medicinal plants containing ursolic acid have been used to treat a wide range of diseases in folk medicine [14]. Although it is a common triterpene, it has shown antibacterial, anti-fungal, anti-inflammatory and anti-mycotic properties as published in recent years [16].

#### 2.2.2. Unknown Pure Compound Analysis

There were 80 fractions collected from the chromatographic procedure using HSCCC. They were all assessed by the use of TLC plates. Fraction 24–28 seemed to be similar and pure on the plate, so these fractions were grouped and sent for NMR analysis.

The NMR analysis suggests compound **2** to be the oroxylin A (Figure 4B). The chemical formula of oroxylin A is C_16_H_12_O_5_, a molecular weight of 284.26 g/mol. The 1H-NMR spectrum showed the most upfield signal at δ 4.05 (O-CH_3_, singlet) related to the methoxy proton while the most downfield signal at δ 12.53 (O-H, singlet) typically refers to a hydroxyl group, such as the one in the position 5 of a flavonoid skeleton. Another two singlets at δ 6.66 and δ 6.73 refer to the position H-8 and H-3, respectively.

The 13C-NMR spectrum shows 15 signals, consisting of eight quaternary carbons, five methines, and one methyl deduced from the DEPT spectrums. The most upfield signal resonated at δ 56.52 is attributed to the methoxy carbon (O-CH_3_), while the most downfield one at δ 182.70 suggests a carbonyl group. The combined spectra data analysis using COSY, HMBC and HSQC, together with the correlation with the literature data, suggests this compound is oroxylin A [17].

Spectroscopic data of oroxylin A: 1H NMR (400 MHz, CDCl_3_): δ(ppm) = 4.05 (3H, s, OCH_3_), 6.66 (1H, s, H-8), 6.73 (1H, s, H-3), 7.57 (2H, m, H3′, H5′), 7.59 (1H, m H′4), 7.94 (2H, dd, *J* = 7.8; 1.5 Hz, H2′, H6′), 12.53 (1H, s, OH); 13C NMR(100 MHz, CDCl3): δ(ppm) = 56.52 (CH3, OME), 90.54 (CH, C-8), 105.48 (CH, C-3), 106.08 (C, C-4a), 126.29 (CH, C-2′, C-6′), 129.12 (CH, C-3′, C-5′), 131.47 (C, C-1′ and C-6), 131.83 (CH, C-4′), 145.62 (C, C-5), 150.77 (C, C-8a), 152.95 (C, C-7), 164.18 (C, C-2), 182.70 (C, C-4). The 1H and 13C NMR spectra can be found in Supplementary Materials.

### 2.3. Quantification of the UA in the Ethanol Extract and Its Fractions in Hexane and Di-Chloromethane by the Use of High-Performance Liquid Chromatography (HPLC)

Solutions of standard ursolic acid were prepared and then injected onto a HPLC system. The HPLC follows the similar condition by Xu et al. [16]. The chromatogram of standard ursolic acid showed a peak with retention time at 13.70 accounting for 98.35% of the total peak area.

A calibration curve was plotted using a range of concentrations of ursolic acid (25–300 μg/mL) against the corresponding peak areas (μV). The equation used was shown as:

Peak area = 28,919 Concentration (R^2^ = 0.9914) (1)

In this way, the concentration of ursolic acid in the ethanol extract and its fractions in hexane and dichloromethane could be obtained as shown in Table 1.

### 2.4. Formalin-Induced Licking Response Test

**Figure 5:**
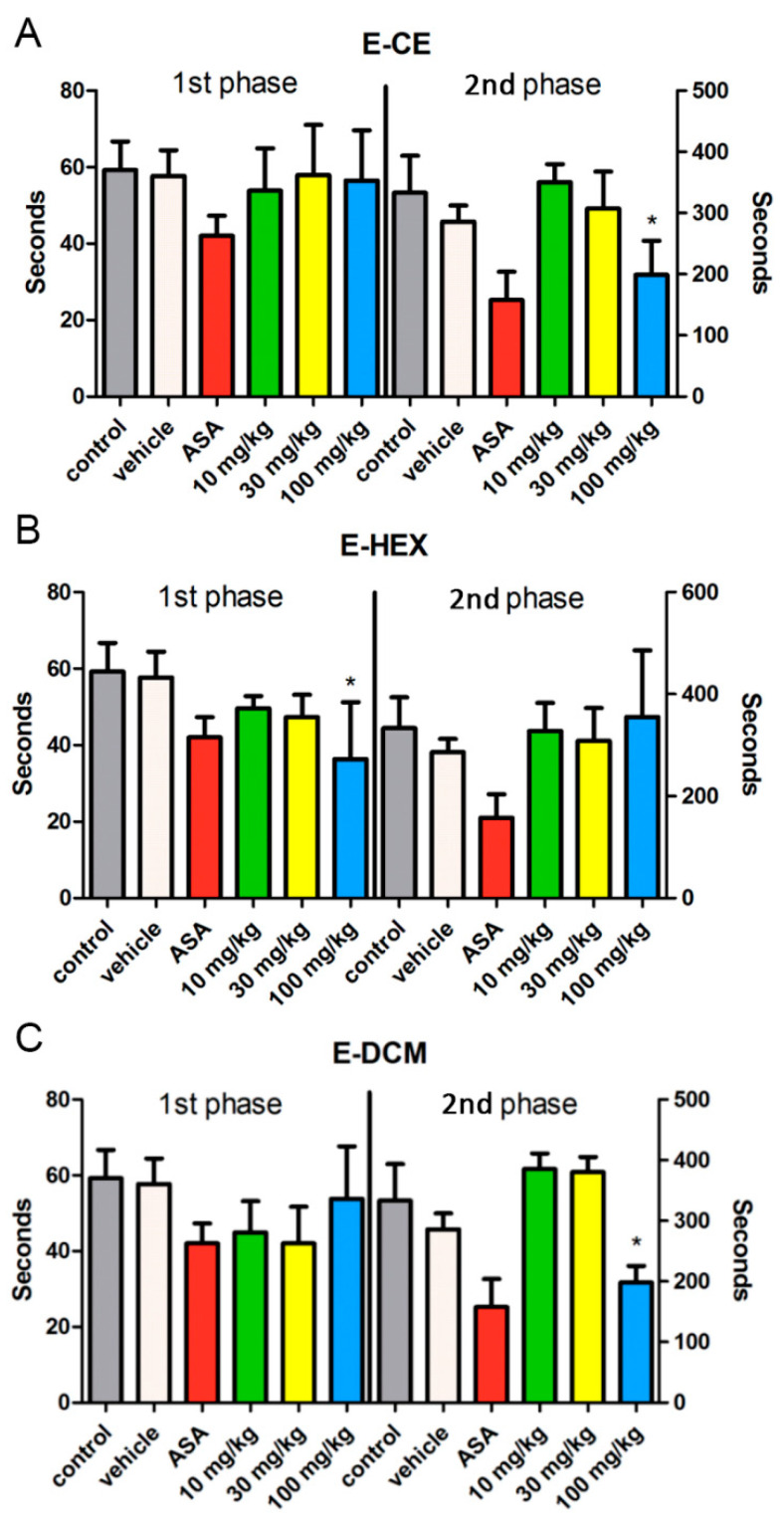
Formalin-induced licking response test for (**A**) crude ethanol extract of *Elsholtzia ciliata* (E-CE), (**B**) fraction in hexane (E-HEX) or (**C**) fraction in dichloromethane (E-DCM) at different concentrations (10, 30 and 100 mg/kg). Animals were pre-treated with an oral dose of acetylsalicylic acid (ASA, 100 mg/kg) or vehicle. The results were presented as mean ± SD (n = 6). Statistical significance between groups (*) was calculated by analyses of variance (ANOVA), followed by Bonferroni’s test. *p* values less than 0.05 (*p* ≤ 0.05) were used as the significant level.

We show here the formalin-induced licking response test to revel the anti-inflammatory activity of the extracts. The response to formalin model shows an early and a late phase. As shown in Figure 5, the crude ethanol extract did not show any activity at the early phase. However, the dose of 100 mg/kg possessed activity in the late one, which suggests anti-inflammatory activity (*p* < 0.0001). The hexane fraction did not show any activity at the late stage. However, the dose of 100 mg/kg possessed activity at the early phase, suggesting antinociceptive activity (*p* < 0.01). The dichloromethane fraction behaved similarly to the crude ethanol extract, with no action in the early phase but with a response in the late stage for the dose of 100 mg/kg, which suggests anti-inflammatory activity (*p* < 0.001).

### 2.5. Central Antinociceptive Activity

**Figure 6:**
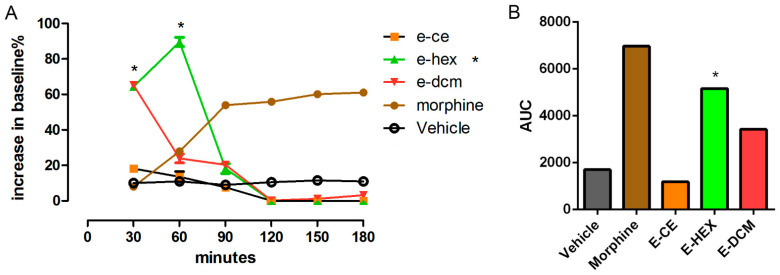
Effect of the crude ethanol extract of *Elsholtzia ciliata* and its fractions in hexane, dichloromethane, ethyl acetate and n-butanol on the hot plate test. Morphine was used as positive control (**A**). The result was also described as area under curve (AUC) of the crude ethanol extract of *Elsholtzia ciliata* and its fractions in hexane and dichloromethane on the hot plate test (**B**). Morphine was used as positive control. Statistical significance between groups (*) was calculated by analyses of variance (ANOVA), followed by Bonferroni’s test. *p* values less than 0.05 (*p* ≤ 0.05) were used as the significant level.

Figure 6A demonstrated a substantial increase in the baseline at 30 and 60 min for the hexane fraction. This increase indicates a potential for this fraction to possess central antinociceptive activity. Mice pre-treated with vehicle did not induce any antinociceptive effect. The morphine-treated animals showed an increase in the baseline 90 min after administration and even a significant increase after 150 min. The results were evaluated statistically by ANOVA test (*p* < 0.001) and comparison with the morphine positive control. We also built an area under the curve (AUC) graphic to better understand these findings (Figure 6B). After the use of the AUC plot, only the hexane fraction shows central antinociceptive activity.

### 2.6. Antioxidant Activity

Table 2 summarizes the results for the DPPH, total phenolic content (TPC) and total flavonoid content (TFC) assays for the crude ethanol extract and its hexane and DCM fractions.

The dichloromethane fraction showed the highest activity with an EC_50_ equivalent value of 0.041 μg/μg. Moreover, the high antioxidant activity of hexane fraction in the DPPH assay is unusual but probably due to non-polar flavonoids, such as Oroxylin A or other phenolics dissolved in the hexane fraction.

The DCM fraction was the one to show the highest content of phenolic compounds with 96.68 ± 0.0010 μg of gallic acid equivalents per mg of the fraction, followed by crude ethanol extract and the hexane fraction.

Once again, the DCM fraction possesses the highest flavonoids content, with 71.50 ± 0.0089 μg of quercetin equivalents per mg.

Flavonoids are reported as compounds that possess high antioxidant activity [18]. The effects are due, but not limited to their capacity to transfer electron free radicals, chelate metal catalysts, activate antioxidant enzymes, reduce alpha-tocopherol radicals and inhibit oxidases.

### 2.7. Antimicrobial Activity

Antimicrobial activity evaluation allowed us to determine the minimum inhibitory concentration (MIC), which is the minimum concentration at which the extract and its fractions were able to inhibit microbial growth in *Escherichia coli*, *Staphylococcus aureus* and *Candida albicans* (Table 3).

Previous studies have already been performed with the essential oil of *Elsholtzia ciliata*. Essential oils from the leaves, flowers and seeds showed antimicrobial activities. The essential oil of the leaves showed the best antimicrobial activities against all tested microorganisms [5].

### 2.8. Cytotoxicity

The crude ethanol extract and its fractions in hexane and DCM were cytotoxic to A549 cell lines (human lung alveolar basal carcinoma epithelial cells); the activity was dose-dependent in 24 and 48 h. The 300 μg/mL concentration of the extract/fractions decreased the cell viability by 100% in 24 and 48 h. For MDA-MB (breast cancer) cell line, the results are similar to the A549 cell line; the extract and fractions also showed cytotoxic activity to MDA-MB cells. The 300 μg/mL concentration of the extract/fractions decreased the cell viability by 98.5% in 24 h and by 97% in 48 h for the hexane extract. The decrease observed for the hexane fraction was 100% in 24 h and 97% in 48 h, and for the dichloromethane fraction, it was by 100% in 24 and 48 h.

## 3. Discussion

This study provides important anatomic and morphological information on *Elsholtzia ciliata* (Thunb.) Hyl. According to this anatomical study, there is little or no difference between the epidermal cells, vascular bundle, palisade parenchyma, spongy parenchyma and collenchyma, while for trichomes and stomata, more attention should be paid for further studies or when carrying out identifications.

The current study on *Elsholtzia ciliata* (Thunb.) Hyl. provides valuable, useful and reliable information on its anatomy. The presence of the three kinds of trichomes, two kinds of stomata and vascular bundle are species-specific and could be used for identification.

From the chemical point of view, ursolic acid and Oroxylin A were isolated from the crude ethanol extract of *Elsholtzia ciliata*. It was the first time Oroxylin A was isolated from this plant. Triterpenes and flavonoids are frequently occurring metabolites in Lamiaceae [19]. They are also sometimes responsible for some of their pharmacological actions.

Formalin and hot plate were used to evaluate the potential of this plant to act as anti-inflammatory or antinociceptive. The response of licking time in the formalin model shows an early and a late phase. The early phase is short-lasting and responded immediately after the injection, which corresponds to neurogenic pain. The late phase is long and persistent, which is associated with the inflammatory response [20]. In the current study, crude ethanol extract and its dichloromethane fraction (100 mg/kg, *p* < 0.05) possessed a significant reduction of the licking time only at the second phase when comparing with the control group; therefore, this inhibition suggested that the action mainly related with the anti-inflammatory activity. Hexane fraction (100 mg/kg) possessed lick-reducing activity only at the early phase. The response suggested nociception related to the peripheral mechanism.

A previous anti-inflammatory study with ursolic acid indicated its ED_50_ value to be 44 mg/kg. Additionally, the nociceptive response was significantly inhibited in rats with the administration of ursolic acid. These results were seen with a dose of 10 mg/kg, both in the neurogenic and the inflammatory phases of the formalin test [21]. In the current study, it is clear to see that crude ethanol extract and its dichloromethane fraction contain a larger quantity of ursolic acid. This finding agrees with the pharmacological results obtained in the formalin test. Both extract and DCM fraction (100 mg/kg) presented significant pharmacological activity at the later phase of this test, which suggest anti-inflammatory activity.

The result of hexane fraction in the hot plate test was in agreement with the formalin test (first phase) that indicated the antinociceptive potential for this fraction. Although hexane fraction showed a higher increase in baseline % than morphine at 30 and 60 min, they were not active throughout the experiment as morphine was, hence the morphine bar was taller than the bars for this fraction. It is also worth mentioning that these results are preliminary. Further studies using receptor antagonists of different pharmacological pathways need to be carried out in the future. These studies will help the understanding of the mechanism of action for the antinociceptive activity related to hexane and dichloromethane fractions and their constituents.

Benincá and collaborators showed in 2011 that ursolic acid was able to inhibit leukocytes migration, IL-1beta, TNF-alpha, MPO and NOx. These results were also seen by Mueller et al., Ku and Lin and Kim et al. who published in 2013 the capacity of ursolic acid in modulating different cytokines in vitro [22].

In this way of using the formalin and the hot plate assays, this study proves the potential this plant has to act as antinociceptive and anti-inflammatory. These are valuable findings as this plant is widely used as an anti-inflammatory TCM in China.

In terms of antioxidant potential, in general, the antioxidant activity as measured by the DPPH assay can be well correlated with the content of phenolics and flavonoids. The results presented by the DPPH assay, total phenolic content and flavonoid equivalents in this study suggest that *Elsholtzia ciliata* possesses high antioxidant activity.

The total phenolic content (TPC), total flavonoid content (TFC), antioxidant activities and anti-inflammatory activities of *Elsholtzia ciliata* have been studied previously [23,24]. Pudziuvelyte et al. found that the amount of phenolic ranged from 61.25 ± 1.91 to 94.67 ± 1.91 mg gallic acid equivalent (GAE)/g dry weight (DW) in the extracts of different parts of the plant, leaf, stem and flower [23]. A similar work performed by Liu et al. showed that the quantity of phenolic compounds in the root’s fraction (497.2 ± 24.9) is higher than those found in the stem’s (213.1 ± 6.2) and inflorescence’s (198.2 ± 10.1) fractions [24]. The content of flavonoids ranged from 5.06 ± 0.08 to 15.43 ± 1.86 mg rutin equivalent (RE) per g/DW with the highest flavonoid level in whole plant and lowest value in the stem. Antioxidant and anti-inflammatory activities were also performed by Pudziuvelyte et al. The DPPH test showed clear evidence of the antioxidant activity of *Elsholtzia ciliata*. The anti-inflammatory activity was assessed by measuring the amounts of inflammatory mediators’ reduction. The results achieved in our study agree with the previous studies by showing a similar trend. The difference in the values of TPC and TFC could be due to the solvent used or conditions during the extraction, the quality of plant materials or environment. Our results confirmed *Elsholtzia ciliata* to be a rich source of phenolic, flavonoid compounds and to possess high antioxidant and anti-inflammatory potential.

The antimicrobial evaluation performed in the current study dealt with the non-volatile extract, named crude ethanol extract and its hexane and dichloromethane fractions. The results showed that the dichloromethane fraction possessed the highest antimicrobial potential against *Candida albicans* (62.5 μg/mL) and some moderate activity against *Staphylococcus aureus* (500 μg/mL). Hexane fraction revealed the highest antimicrobial potential against *Escherichia coli* (250 μg/mL).

As reported by Dorman and Deans (2000) phenolic compounds, such as flavonoids, may be responsible for the antimicrobial activity of plant extracts [25]. The specific mechanism involved here is the destabilization of the cytoplasmic membrane by compounds with phenolic groups (OH bound to aromatic rings), which act as proton exchangers, reducing the pH gradient across the cytoplasmic membrane and thus leading to cell death.

The antimicrobial results suggested that the non-volatile (extracts) components also showed potential to be exploited as effective antimicrobial medicines.

The cytotoxicity test with two cancer cell lines showed that the higher activity is observed in 24 h and that the 300 μg/mL concentration of the crude ethanol extract and its fractions decreased the cell viability more than the other doses. The ethanol extract and its fractions showed an anti-proliferative effect on cells except for the concentrations of 10, 100 μg/mL for the hexane and dichloromethane fractions. Generally, a positive correlation was seen between the cell viabilities and the used extract concentrations. The results determined the positive effects of the extract and its fractions on cell viability mainly up to 24 h.

Ursolic acid in synergy with other constituents in the extract and its fractions can be responsible for the observed cytotoxic effect. Gu et al. in 2012 showed that ursolic acid was able to inhibit MCF-7 cells proliferation, increasing p53 and p21WAF1/Cip1 expression. Zhao et al., in 2013, also saw for the same cell type MCF-7 that ursolic acid was able to induce apoptosis of the cells through upregulation of MCL1 and autophagy by MAPK1/3 pathway [26].

On the other hand, Oroxylin A inhibited the viability of hepatocarcinoma cell line HepG2 by substantially producing H_2_O_2_ intracellularly and activating the PERK-eIF2-ATF4-CHOP branch of the unfolded protein response (UPR) pathway. These mechanisms resulted in the induction of TRB3 and causal reduction of p-AKT1/2/3 (Ser473) (Xu et al., 2012) [27].

In the present study, we isolated Oroxylin A from the Hexane fraction, which can contribute for the overall cytotoxic potential of this plant.

## 4. Materials and Methods

**Plant materials.** Leaves of *Elsholtzia ciliata* (Thunb.) Hyl collection (900 g) took place in Jiangxi Province, China, in August 2013. Part of the material suffered herborization. Dr. Hua Yang identified the plant. A voucher number: CPU-2013101802, is registered in the herbarium of the China Pharmaceutical University (CPU).

**Animals.** This study used Swiss Webster mice (20–25 g), donated by Instituto Vital Brazil (Niteroi, Rio de Janeiro, Brazil). A room with a light-dark cycle of 12 h, 22 ± 2 °C, 60% to 80% humidity and with food and water provided ad libitum were the conditions adopted for the animals. We used mice only one time per experiment after acclimatisation to the laboratory conditions for at least one hour before the beginning of each test. All protocols were conducted per the Guidelines on Ethical Standards for Investigation of Experimental Pain in Animals (Zimmermann, 1983) [28] and followed the principles and guidelines adopted by the National Council for the Control of Animal Experimentation (CONCEA), approved by the Ethical Committee for Animal Research (# DFBCICB015–04/16, 31/19 and 34/19). All experimental protocols only took place during the light phase. Each group used a minimum number of animals, and at the end of each experiment, we used a ketamine/xylazine overdose to sacrifice the animals.

**Cell culture.** Human lung alveolar basal carcinoma epithelial cells (A549) and MDA-MB breast cancer cells were cultured at 37 °C under 5% CO_2_ for 2–3 days in an incubator in RPMI medium supplemented with 10% fetal bovine serum and 1% antibiotics (penicillin and streptomycin). The cells were then sedimented by centrifugation at 1200 rpm for 5 min. After removing the supernatant, the cell pellets were resuspended in a fresh medium.

**Microorganisms.** This study used three strains of microorganisms, one Gram-positive, one Gram-negative and one fungus. Culture conditions used: *Staphylococcus aureus* MRSA (BMB9393) and *Escherichia coli* were clinically isolated from the Clementino Fraga Filho Hospital, UFRJ, Brazil. The bacteria were cultured under the condition of Brain Heart Infusion Agar cattle (BHI) for 24 h at 37 °C. *Candida albicans* serotype B ATCC 36802 was originally from the Federal University of São Paulo, Brazil and cultured on Sabouraud Agar for 48 h at room temperature.

**Reagents and chemicals.** Sodium hypochlorite, acetic acid, hydrogen peroxide, dimethyl sulfoxide, deuterated chloroform, acetylsalicylic acid (ASA), 2,2-Diphenyl-1-picrylhydrazyl (DPPH), Folin–Ciocalteu phenol reagent, aluminium trichloride anhydrous, gallic acid 3-[4,5-dimethylthiazol-2-yl]-2,5 diphenyl tetrazolium bromide (MTT) were purchased from Sigma–Aldrich Chemical Co. Sodium carbonate anhydrous was obtained from BDH Chemicals Ltd. Poole England. Formalin was purchased from Merck Inc. Morphine was provided by Cristália (São Paulo, Brazil). Quercetin, hexane, dichloromethane, ethyl acetate, ethanol, methanol, chloroform and n-butanol were obtained from Trinity College Dublin Hazardous Materials Facility solvent store.

**Anatomic study.** Cross-sections were manually performed on leaves and stems using commercial razor blades. Sections were clarified with sodium hypochlorite 20%, neutralized with acetic acid 0.2%, washed in distilled water and stained with a solution of Astra blue and Safranin [29]. The dissociation section was prepared by making fragments in a solution of hydrogen peroxide and acetic acid (1:1), washing in distilled water and staining with Safranin 50% [30]. The sections were mounted with glycerinated gelatin 50%. The images were obtained using a photomicroscope Leica DM750 with the image capture system ICC50 HD and Qwin program.

**Preparation of plant extracts and the isolation of bioactive compounds.** The crude ethanol extract was prepared by continuous hot extraction of the plant material (900 g) using soxhlet for 72 h. A liquid–liquid extraction of the evaporated total ethanol extract, suspended in water, was executed to obtain the following fractions of increasing polarities: hexane, dichloromethane, ethyl acetate and n-butanol.

In a parallel protocol, the crude ethanol extract was also chromatographed using counter-current chromatography. The HSCCC separation conducted on an IntroPrep TM (Quattro) counter-current chromatography (AECS, Bridgend, UK) equipped with one bobbin containing one polytetrafluoroethylene multi-layer coils (136 mL, 2.0 mm inside diameter); rotation speed at 850 rpm and the column axis horizontal had a flow of 2 mL per minute. A constant flow pump Series II (Scientific Systems Inc., Lab Alliance, Prague, Czech Republic) was connected to the HSCCC system. Four mL fractions were manually collected at an interval of 2 min. The sample was injected into the 95 mL coil using a 5 mL sample loop. The selected solvent system was a classic HEMWat (hexane:ethyl acetate:methanol:water), and the ideal ratio of each solvent was 4:6:5:5. The mobile phase was the upper phase (organic) the stationary phase was the lower phase (aqueous). The sample was dissolved in the selected solvent system and applied to the HSCCC apparatus through a 5 mL loop. The mobile phase was pumped at 2 mL per minute until the tube was 50, the rotation and the pumping of the mobile phase stopped, and we started extrusion. Thin layer chromatography (TLC) was the technique of choice to analyse all the fractions collected from HSCCC, with the mobile phase of chloroform, methanol and water (9:1:1, v/v/v). We then performed the combination of all fractions with a similar pattern on TLC.

**High performance liquid chromatography (HPLC) system.** The quantification of possible bioactive compounds was performed by the use of a high-performance liquid chromatography (HPLC) system. Standard ursolic acid was purchased from Sigma-Aldrich Ireland Limited (Arklow, Co., Wicklow, Ireland). Standard solutions were prepared in methanol at different concentrations (300, 250, 200, 150, 100, 50 and 25 µg/mL). A volume of 20 µL of each solution was injected in four replicates to allow the plot of a calibration curve. The HPLC system (Waters^®^) used was comprised of Waters 1525 binary HPLC pump, Waters 2487 dual λ absorbance detector (at λ = 215 nm), Waters 717 plus auto-sampler and breeze software. The column was a Thermo C18 column (250 × 4.6 mm, 5 μm). The mobile phase was a mixture of methanol and distilled water (95:5, v/v). A filter (Nylon66) 0.45 mm was used to filtrate the samples. A methanol solution of each extract was prepared at a final concentration of 500 µg/mL. The condition selected was an isocratic elution with a volume of 0.4 mL/min.

**Formalin-induced licking test.** The method used in the formalin test was similar to the methodology of Hunskaar and Hole (1987) and Gomes et al., (2007) [31,32]. Crude ethanol extract and its fractions were dissolved in Dimethylsulfoxide (DMSO) at a concentration of 100 mg/mL, then diluted according to the weight of mice at doses of 10, 30, 100 mg/kg in PBS. One hundred microlitres of each sample were given through oral gavage, and after one hour, 20 μL of formalin was injected in the right hind paw. Mice, pre-treated with Acetylsalicylic acid (ASA), were used as a positive group. The control group was composed of the mice only injected by formalin, while the vehicle group was composed of the ones pre-treated with PBS. The licking time response at the early phase (Phase I, 0–5 min) suggested analgesic effects, while the late one (Phase II, 15–30 min) suggested anti-inflammatory effects.

**Hot plate test.** The hot plate test followed the methodology described by Matheus et al., (2005) [33]. All samples, extracts and fractions were tested for central neuropathic antinociceptive activity. A Group of mice (n = 5) were placed on a hot plate (Insight Equipment, Rio de Janeiro, Brazil) at a temperature of 55 ± 1 °C. The same concentrations of extracts as the ones used for the formalin test (10, 30, 100 mg/kg) were administered through oral gavage. The time spent licking and flicking their paws (reaction time) was recorded at intervals of 30 min. Morphine (5 mg/kg) was used as the positive control. The baseline is defined by the mean reaction time of the animal in the hot plate at 60 and 30 min before extract administration.

There are two ways to quantify the anti-nociception, one is increase in baseline (%), and another one is area under curve (AUC).

Increase in baseline (%) = [(reaction time × 100)/base line] − 100(2)

AUC = 30 × IB [(min 30)/2 + (min 30 + min 60)/2 + (min 60 + min 90)/2 + … + (min 180)/2](3)

where IB = Increase in baseline (%).

**Antioxidant Activity.** The antioxidant activity of the extracts was by the use of the scavenging free radical potency using the DPPH, the total phenolic content determination and the total-flavonoids content determination. The DPPH assay followed the methodology described by Mensor et al., (2001) [34]. The total phenolic assay used the Folin–Ciocalteu reagent according to the method described by Gursoy et al., (2009) [31]. The determination of flavonoid content of the extracts followed the methodology described by Gursoy et al., (2009) [35]. All three techniques suffered adaptations with modifications to microscale based on the description by Ramos and Boylan (2010) [36].

**Antimicrobial activity.** The crude ethanol extract and its fractions were dissolved in DMSO 2% at the concentration of 100 mg/mL and 20 mg/mL; they were then diluted in Mueller–Hinton broth (for bacteria) and RPMI-MOPS (for fungi, pH = 7.2). The pure culture medium was the negative control group, while the medium inoculated with bacteria/fungi with gentamicin (for bacteria)/amphotericin B (for fungi) were the positive control groups.

For bacterial inoculum, 10 μL of inoculum and 100 μL Mueller-Hinton medium with extracts were added to 96-well plates. One hundred μL of inoculum and 200 μL RPMI medium with extracts were added to 96-well plates for fungal inoculum. The final concentration of both fungal and bacterial in each well was 5 × 10^5^ CFU/mL. After incubation for 24 h at 37 °C, the MIC was determined visually by turbidity after adding the microbial stain—resazurin (0.005% in PBS, pH 7.2). The resazurin (blue) oxidises to the resorufin (pink) in the presence of viable cells.

**Cytotoxicity by the use of MTT test.** Human lung alveolar basal carcinoma epithelial cells (A549) and MDA-MB breast cancer cells received treatment with each extract and fractions (samples) at the concentrations of 1, 10, 30, 100 and 300 µg/mL for 24 and 48 h. One hundred µL of the RPMI medium containing 0.5 mg/mL MTT was added after removing the supernatants. The formed MTT-formazan crystals were dissolved in DMSO and had their absorbance measured on a FlexStation microplate reader at 540 nm. The control group—considered 100% of cell viability—consisted of the medium and cells, with no sample.

**Statistical analysis.** All experimental groups were performed in triplicate. All experimental groups with mice used six animals per assay. Data were analysed using GraphPad Prism 5.0 software (results described as mean ± SD). The IC_50_ values were obtained by linear regression and showed an acceptable coefficient of determination (R^2^ ≥ 0.90). Statistical significance between groups was calculated by analyses of variance (ANOVA), followed by Bonferroni’s test. *p* values less than 0.05 (*p* ≤ 0.05) characterized a significant level.

## 5. Conclusions

The current study with *Elsholtzia ciliata* (Thunb.) Hyl. leaves provided valuable, useful and reliable information on this plant’s pharmacognostic evaluation with botanical, chemical and pharmacological basis for further and more intensive studies.

*Elsholtzia ciliata* contained characteristic anatomic features consistent with Lamiaceae plants and the description of its anatomy would help in the quality control of this herb. The combined species-specific characteristic of stomata, vascular bundle and trichomes could be used for identification purposes.

Ursolic acid was identified and quantified in all fractions. There is a correlation between its quantity and the preliminary anti-inflammatory activity for each extract tested. Oroxylin A was isolated from the leaves of this herb by the use of HSCCC.

Fractions of this plant possess peripheral antinociceptive and anti-inflammatory activities as well as central antinociceptive activity. This is an important finding as this plant is widely used as an anti-inflammatory TCM in China.

The fractions also showed moderate antimicrobial activity for both bacteria and fungi, high antioxidant activity and cytotoxic potential against two cancer cell lines. These results together confirm the potential of this TCM for the activities that are described popularly but more importantly it provides the basis for future quality control analyses based on its chemical and botanical evaluations.

## Figures and Tables

**Table 1:** The concentration of ursolic acid in each extract and fractions.

Group	Concentration of Ursolic Acid (μg/mL)			Average (μg/mL)
Crude ethanol	35.10	37.62	37.30	36.67 ± 1.37
Hexane	4.66	4.62	4.55	4.61 ± 0.06
Dichloromethane	32.30	27.20	33.30	30.93 ± 3.27

**Table 2:** EC_50_ values, EC_50_ equivalent, total phenolic content and total flavonoid content of the crude ethanol extract of *Elsholtzia ciliata* and its fractions in hexane, dichloromethane, ethyl acetate and n-butanol.

Extract and Partitions	EC_50_ Equivalent (μg/μg DPPH)	Total Phenolic Content (μg GAEs/mg Extract) *	Total Flavonoid Equivalent (μg QEs/mg Extract) *
Crude ethanol	0.15	38.32 ± 0.0010	9.45 ± 0.0035
Hexane	0.46	38.32 ± 0.0012	0
Dichloromethane	0.041	96.68 ± 0.0010	71.5 ± 0.0089

* GAEs—Gallic acid equivalents, Qes—Quercetin equivalents.

**Table 3:** Antimicrobial activity of crude ethanol extracts and its fractions (non-volatile component).

Microorganisms	Amphotericin B	Gentamicin	Crude Ethanol	Hexane	Dichloromethane
*Escherichia coli*	-	10 μg/mL	1000 μg/mL	250 μg/mL	1000 μg/mL
*Staphylococcus aureus*	-	10 μg/mL	-	1000 μg/mL	500 μg/mL
*Candida albicans*	0.19 μg/mL	-	-	-	62.5 μg/mL

## Data Availability

Data is contained within the article.

## Supplementary Materials

The following are available online at https://www.mdpi.com/article/10.3390/ph14111152/s1, Figure S1: The 1H NMR spectrum of Oroxylin A in CDCl_3_, Figure S2: The 13C NMR spectrum of Oroxylin A in CDCl_3_.
