# Supplementary material for: Pharmacognostic Study on Elsholtzia ciliata (Thumb.) Hyl: Anatomy, Phytochemistry and Pharmacological Activities
Source: Pharmaceuticals (Basel). 2021 Nov 12;14(11):1152. doi: 10.3390/ph14111152 (PMC8620380; doi:10.3390/ph14111152)
Supplement: Supplementary file 1 [file pharmaceuticals-14-01152-s001.zip › pharmaceuticals-1457278-supplementary.pdf]

Supplementary Information for

Pharmacognostic Study on Elsholtzia ciliata (Thumb.) Hyl: Anatomy, Phytochemistry and Pharmacological Activities

Qian Zhang [1, 2,*], Niara Moura Porto [3], Carolina Carvalho Guilhon [4], Thais Biondino Sardella Giorno [4], Daniela Sales Alviano [5], Maria de Fatima Agra [3], Patricia Dias Fernandes [4] and Fabio Boylan [1,*]

[1]School of Pharmacy and Pharmaceutical Sciences, Trinity Biomedical Sciences Institute, Trinity College Dublin, Dublin 2, Ireland

[2]Intelligent Sensing and AI Center, National University of Singapore (Chongqing) Research Institute, Chongqing, 401123, China

[3]Laboratory of Taxonomy and Pharmacobotany, Federal University of Paraíba, João Pessoa, 58051-970, Brazil; niaraporto@gmail.com (N.M.P.); agramf@ltf.ufpb.br (M.d.F.A.)

[4]Laboratory of Pharmacology of Pain and Inflammation, Institute of Biomedical Sciences, Federal University of Rio de Janeiro, Av. Carlos Chagas Filho 373, bloco F. Prédio novo ICB, Rio de Janeiro, 21941-901, Brazil; carolguilhon@gmail.com (C.C.G.); thais.sardella.farma@hotmail.com (T.B.S.G.); patricia.dias@icb.ufrj.br (P.D.F.)

[5]Department of Microbiology, Federal University of Rio de Janeiro, Rio de Janeiro, 21941-901, Brazil; danialviano@gmail.com

*Authors to whom correspondence should be addressed: zhangqi@tcd.ie (Q.Z.); fabio.boylan@tcd.ie (F.B.)

S1. NMR analysis of unknown pure compound.

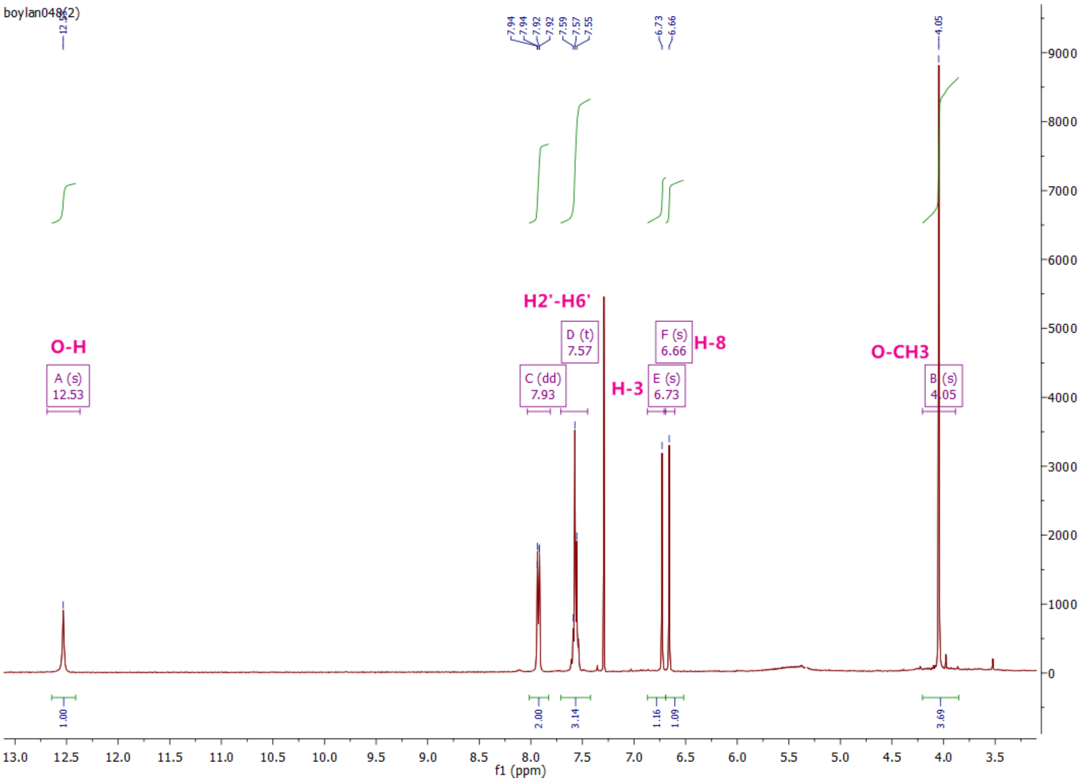

Figure S1. The ^{1}H NMR spectrum of Oroxylin A in $CDCl_3$.

The chemical shifts of the detected proton peaks are listed below:

δ1H NMR (400 MHz, $CDCl_3$) δ12.53, 7.94, 7.92, 7.59, 7.57, 7.55, 6.73, 6.66, 4.05.

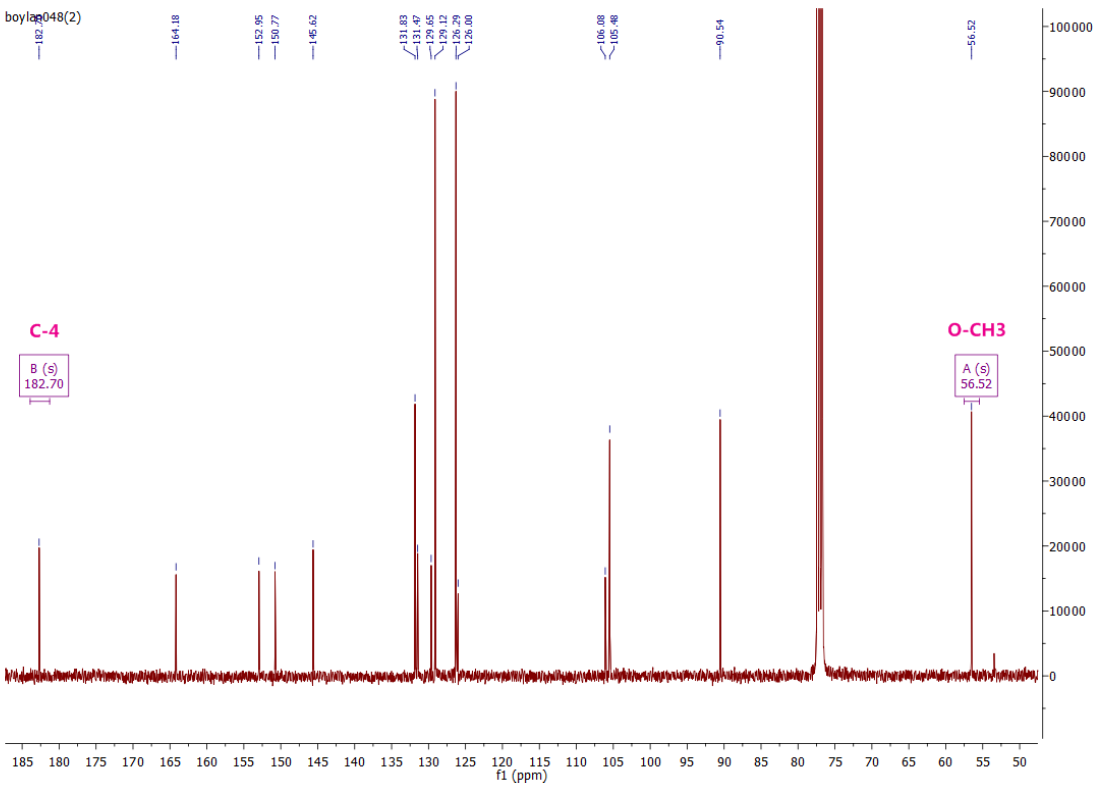

Figure S2. The 13C NMR spectrum of Oroxylin A in $CDCl_3$.

A total of 15 carbons between 50-185 ppm are shown in the 13C-NMR spectrum:

13C NMR (100 MHz, $CDCl_3$) δ182.70, 164.18, 152.95, 150.77, 145.62, 131.83, 131.47, 129.65, 129.12, 126.29, 126.00, 106.08, 105.48, 90.54, 56.52
